# Exploring specific primers targeted against different genes for a multiplex PCR for detection of *Listeria monocytogenes*

**DOI:** 10.1007/s13205-014-0225-x

**Published:** 2014-05-21

**Authors:** Ashwani Kumar, Sunita Grover, Virender Kumar Batish

**Affiliations:** 1 Molecular Biology Unit, Dairy Microbiology Division, National Dairy Research Institute, Karnal, 132001 Haryana India; 2 Department of Biotechnology, Seth Jai Parkash Mukand Lal Institute of Engineering and Technology, Radaur, Yamuna Nagar, 135133 Haryana India

**Keywords:** Primers, Evaluation, Multiplex PCR, *Listeria monocytogenes*, Detection, Dairy foods

## Abstract

The efficacy of six different sets of primers targeted against *16S rRNA* and virulence genes such as ‘*iap*’, ‘*hly*’ and ‘*prf*’ was evaluated in separate PCR assays. The primer pairs targeted against *16S rRNA* resulted into amplification of 1.2 kb PCR product. However, sets of primers targeted against different regions of ‘*iap*’ produced 371 and 660 bp PCR products, respectively. The primer pair targeted against ‘*prf*’ gene could produce 508 bp product. Three primer pairs targeted against different regions of ‘*hly*’, i.e., ‘*hly*’, ‘*hly A*’ and ‘*hly K9*’ were able to amplify 713, 276 and 384 bp products, respectively. The PCR conditions were also optimized in respect of two internal sets of primers falling within ‘*iap*’ and ‘*hly*’ genes that amplified 119 and 188 bp products to verify the PCR results obtained with respective external sets of primers. Three different combinations involving four sets of primers based on *16S rRNA*, ‘*iap*’, ‘*hly*’ and ‘*prf*’ were explored in respective multiplex PCR assays in order to select a suitable combination. Combination 1 and 3 worked successfully as revealed by amplification of all the four bands of expected sizes on agarose gel. However, while optimizing the different parameters for developing a functional multiplex PCR, it was observed that in both these combinations, only two of the amplified products, i.e., 1.2 kb and 713 bp could be invariably detected. Hence, these two primers were combined in the multiplex PCR and the conditions were optimized for application in dairy foods for detection of *Listeria monocytogenes.*

## Introduction

*Listeria monocytogenes*, a high-risk emerging food pathogen, has recently assumed lot of interest as a result of its association with several outbreaks of listeriosis across the world through implication with wide variety of foods, both raw and processed (Dalton et al. 1997; CDC 2000; Kumar et al. 2012, 2014). The reports available in India regarding the incidence of *L. monocytogenes* have been analyzed by many workers (Khan et al. 2011; Vinothkumar et al. 2013; Trimulai 2013). Moreover, the occurrence of *L. monocytogenes* in India has been underreported in many cases because of the inefficient surveillance and monitoring system. The ability of this emerging food pathogen to survive and grow in many foods during processing and storage has been attributed to its ubiquitous nature, resistance to diverse environmental conditions such as low pH and high salt concentrations and its microaerobic and psychrotrophic nature. The psychrotrophic nature to grow and survive at a wide range of temperature (2–40 °C) in or on foods for prolonged periods under adverse conditions has made *L. monocytogenes* a major concern for the agri-food industry during the last decade. Mandatory compliance issued by food and drug administration for zero tolerance ruling for this organism in processed/ready-to-eat foods has emphasized the need for development of molecular-based rapid methods for detection of *L. monocytogenes*.

Keeping in view the limitations associated with conventional, immunological and nucleic acid probe assays, several PCR-based formats have been evolved for detection of *L. monocytogenes* (Kumar et al. 2012). In this context, the gene cassette along with ‘*iap*’ gene involved in pathogenicity of *L. monocytogenes* has been made real targets for its rapid detection by means of PCR-based assays. The expression of three different *L. monocytogenes* virulence genes (*iap*, *hly* and *prf* A) was examined by several investigators to determine the suitable target for specific DNA amplification using gene-specific primers. The ‘*iap*’ gene encodes for a 60 kDa basic extracellular protein (p60) which acts as murein hydrolase involved in septum formation (Wuenscher et al. 1993). The exact role of p60 in invasion is, however, still not clear. Another virulence gene hemolysin encodes two virulence factors involved in the lysis of vacuole namely listerolysin O (LLO) and phosphatidylinositol-specific phospholipase C (PI-PLC) by means of pore-forming activity and hydrolysis of glycophosphatidyl inositol anchors, thereby, leading to escape of organisms from the vacuole in primary macrophages (Portnoy et al. 1992; Sheehan et al. 1994). The use of *16S rRNA* gene as a distinct signature for a bacterial species has become the method of choice for identifying and differentiating microorganisms because of multiple copies (10^4^) of rRNA present in cell (Wang et al. 1992). Based on *16S rRNA* gene sequences, several PCR assays have been developed for detection of *Listeria monocytogenes* at both genus and species level (Wiedmann et al. 1993; Czajka et al. 1993). The virulence gene ‘*hly A*’ has also been targeted by different investigators for the development of PCR-based assays intended for detection of *L. monocytogenes* (Deneer and Boychuk 1991; Fluit et al. 1993; Norton and Batt 1999). The ‘*iap*’ gene common to all members of the genus Listeria had also been chosen as a suitable target after finding that there were conserved gene portions at 5′ and 3′ ends, while internal portions are highly specific (Bubert et al. 1992). In this investigation, we evaluated the efficacy of a few selected pair of primers individually and in different combinations with the objective of developing a reliable Multiplex PCR for detection and identification of *L. monocytogenes*.

## Materials and methods

### Bacterial cultures and their maintenance

The bacterial cultures used in this investigation included pathogenic strains of *L. monocytogenes* along with other cultures. *Listeria monocytogenes* ATCC 7644 was purchased from Thermo Scientific, UK and *L. monocytogenes* Scott A was procured from DM Division, NDRI, Karnal. The cultures used in this study were propagated in BHI (brain heart infusion)/TSB (Trypticase soya broth) at 37 °C for 18 h. The cultures were preserved on BHI/Trypticase soya agar slants and stored in refrigerator or as glycerol stocks stored at −70 °C ultra low deep freezer (New Brunswick Scientific, USA) until further use. The cultures were activated in BHI broth prior to use by sub-culturing at biweekly intervals.

### Preparation of template DNA

#### Broth cultures

The template/genomic DNA was prepared from broth cultures of *Listeria monocytogenes* by following boiled lysate method (Witham et al. 1996) as well as the method of Pospiech and Neikmann (1995). The boiled lysate was prepared by harvesting the overnight grown culture of the test organism followed by heating the bacterial suspension in 50 MilliQ water for 5 min in a boiling water bath and then centrifuging for 5 min at 10,000 rpm to separate the supernatant containing DNA. For Pospeich and Neikmann’s method, the cells were harvested from one ml of overnight grown cultures of *L. monocytogenes* and resuspended in 0.5 ml of SET buffer (75 mM NaCl, 25 mM EDTA, 20 mM Tris) and lysozyme was added at a concentration of 1 mg/ml (25 mM Tris, lysozyme, 10 mg, 5 M NaCl) followed by incubation at 37 °C for 1 h. The subsequent step was the addition of 1/10th volume of 10 % SDS and 0.5 mg/ml of proteinase K and incubation further continued for 2 h at 55 °C. One-third volume of 5 M NaCl and one volume of chloroform were added and incubated at room temperature for 30 min with frequent inversions. The samples were centrifuged and upper aqueous phase transferred to a new tube and the DNA was precipitated by adding one volume of isopropanol or two volumes of ethanol. The DNA was pelleted, dried and dissolved in TER buffer containing 10 μg/ml of RNase A.

### PCR assay

The PCR amplification for detection of *Listeria monocytogenes* was performed using Eppendorf master cycler gradient, 5331, Germany. The selected oligonucleotide primers for detection of *Listeria monocytogenes* were got custom synthesized (Bangalore Genei, India). The description of the primer pairs used in this study is given in Table 1. The PCR assay was performed in 25 µl reaction mixture comprising of 100 ng of template DNA, 10× PCR buffer (containing MgCl_2_), 0.2 mM (each of primers), 0.2 mM (each) dNTPs and 1 unit of Taq polymerase (Boehronger Mannheim). Appropriate positive and negative controls with each reaction were also set up. The PCR cycling parameters used for each set of primers are as per the published literature and will be described in the “Results and Discussion”.

**Table 1:** Description of the primers used in the present investigation

S. no.	Target gene	Primers	Primer sequence	Size of amplified product (bp)	References
1	*16S rRNA*	Lm3	5′-ggACCggggCTAATACCgAATgATAA-3′	1,200	Wiedmann et al. (1993)
		Lm5	5′-TTCATgTAggCgAgTTgCAgCCTA-3′		
2	*iap*	ELMIAPF	5′-CAAACTgCTAACACAgCTACT-3′	371	Klein and Juneja (1997)
		ELMIAPR	5′-gCACTTgAATTgCTCTTATTg-3′		
		Mono A	5′-CAAACTgCTAACACAgCTACT-3′	660	Bubert et al. (1999)
		Lis 1B	5′-TAATACgCgACCgAAgCCAAC-3′		
3	*Hemolysin*	Hly 1	5′-ATTTTCCCTTCACTgATTgC-3′	276	Cooray et al. (1994)
		Hly 2	5′-CACTCAgCATTgATTTgCCA-3′		
		ELMHLYF	5′-TCCgCCTgCAAgTCCTAAgA-3′	713	Klein and Juneja (1997)
		ELMHLYR	5′-gCgCTTgCAACTgCTCTTTA-3′		
		ILMHLYF	5′-gCAATTTCgAgCCTAACCTA-3′	188	Klein and Juneja (1997)
		ILMHLYR	5′-ACTgCgTTgTTAACgTTTgA-3′		
		HF9	5′-gTTTggTTAATgTCCATgTT-3′	384	Wagner et al. (2000)
		HR9	5′-TATTCTAgTCCTgCTgTCCC-3′		
4	*prf A*	ELMPRFF	5′-CgggATAAAACCAAAACAATTT-3′	508	Klein and Juneja (1997)
		ELMPRFR	5′-TgAgCTATgTgCgATgCCACTT-3′		

### Multiplex PCR assay

For developing a multiplex PCR assay for detection of *L. monocytogenes* in foods, four sets of different primers evaluated previously were tried in three different combinations simultaneously in one assay.

### Optimization of multiplex PCR amplification conditions

Amplification conditions were optimized with respect to annealing temperature (Gradient PCR using 60 °C with a gradient of 2 °C), Taq polymerase concentration (0.5–3.0 units), MgCl_2_ concentration (1 mM–3.0 mM), Primer concentration (25 ng–100 ng), annealing time (30, 45 and 60 s), extension time (30 s and 1 min) and number of cycles (25, 30, 35 and 40) for the four sets of primers used in the multiplex PCR assay.

### Analysis of PCR products

The PCR amplified products were electrophoresed on 2 % agarose gel containing 0.5 µg/ml of ethidium bromide. The gel was visualized under UV transilluminator and photographed using Polaroid 667 packfilm with MP4 system polaroid camera (Photodyne, USA). The molecular size marker consisted of 100 bp DNA ladder comprising of 100–1,000 bp bands (Bangalore Genei, India).

## Results and discussion

### Evaluation of primers for *Listeria monocytogenes*

**Fig. 1:**
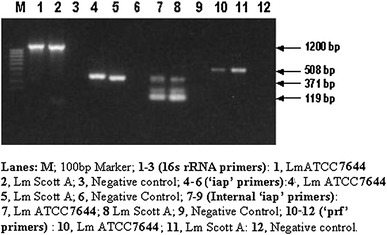
Evaluation of different sets of primers targeted for detection of *Listeria* and *Listeria monocytogenes* by PCR amplification

**Fig. 2:**
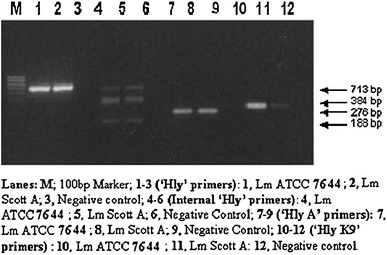
Evaluation of primers using different regions of hemolysin gene of *Listeria monocytogenes*

During the initial part of this study, we tested the efficacy of six different sets of primers targeted against *16S rRNA* (genus specific) and virulence genes such as ‘*iap*’, ‘*hly*’ and ‘*prf*’ *(L. monocytogenes* specific) in their respective PCR assays using common PCR cycling parameters as their annealing temperatures were pretty close. These include initial denaturation at 95 °C for 4 min followed by 35 cycles each of denaturation at 94 °C for 30 s, annealing at 60 °C for 1 min and extension at 72 °C for 1 min and the final extension of 72 °C for 5 min. The results pertaining to the amplification of the *Listeria monocytogenes*-specific template DNA extracted by Pospiech and Neikmann's method/boiled lysate with individual primers pairs have been presented in Figs. 1 and 2.

### Genus-specific primers for *Listeria* spp.

#### 16S rRNA-based primers Lm3/Lm5

*16S rRNA* has been targeted in the identification of a number of bacteria both at genus and species level by exploring the conserved and variable regions of the gene. (Gopo et al. 1988; Maureau et al. 1989). The choice for targeting *16S rRNA* gene has been dictated by the presence in microorganisms of multiple copies (10^4^) of rRNA, thereby, increasing the ease of signal generation of the assays. Specific DNA probes or PCR primers have been designed from variable ribosomal RNA regions and used for the detection of specific target cells, e.g., the detection of *L. monocytogenes* (Wang et al. 1991), Aeromonas (Barry et al. 1990), Lactic acid bacteria (Klijn et al. 1991) and Salmonella species (Lin and Tsen 1996). Prompted by the advantages offered by 16S rRNA, we also targeted this gene in our study for exploring a set of primers based on the *16S rRNA* for determining its suitability in a PCR assay for detection of *Listeria.*

The first primer targeted against *16S rRNA* included in this study was intended for detection of all types of Listeria at genus level. A PCR assay was standardized using the primer pair in combination with template DNA from two of the *Listeriamonocytogenes* strains ATCC and Scott A. An amplified PCR product of 1,200 bp was detected on the agarose gel with template from both the strains (Fig. 1, Lanes 1 and 2). The PCR amplification conditions include initial denaturation at 95 °C for 4 min followed by 35 cycles each of denaturation at 94 °C for 30 s, annealing at 60 °C for 1 min and extension at 72 °C for 1 min and the final extension of 72 °C for 5 min. The primer pair appears to be highly specific for *Listeria* only at these amplifications parameters, as no specific bands could not be observed.

Our results in this regard are consistent with the earlier findings of Wiedmann et al. (1993) who also achieved the amplification of 1.2 Kb fragment with the help of primers Lm3 and Lm5 in their PCR assay used in conjunction with LCR with all *Listeria*spp. except *L. grayi*. This assay was based on a single base pair difference in the V9 region of the sequence of the genes coding for ribosomal RNA which distinguished *L. monocytogenes* from other closely related *Listeria*spp. The results from our study clearly demonstrate that the two PCR primers used in the PCR assay were genus specific since the 1.2 Kb amplicon could not be detected in any other organism other than *Listeria*.

*16S rRNA* was also explored previously (Wang et al. 1991, 1992) for detection of *Listeria monocytogenes* in foods spiked with the target organism. They used pair of primers based on a unique region in the *16S rRNA* sequence in *L. monocytogenes* to yield a specific nucleic acid probe. The method was found to be extremely sensitive as it could detect as low as 2–20 cfu/ml of *L. monocytogenes* in pure cultures and as few as 4–40 cfu in inoculated diluted food samples.

### Species-specific primers targeted against virulence genes of *L. monocytogenes*

The pathogenicity of *L. monocytogenes* is associated with a number of virulence factors which are encoded on a multigene family common to all *Listeria monocytogenes* strains. Some of these virulence genes could also be very attractive candidates for targeting in the development of PCR-based assays for detection of *L. monocytogenes*. In this study, we evaluated five sets of primers based on ‘*iap*’, ‘*hly*’ and ‘*prf*’ genes in their respective PCR assays. The results pertaining to the suitability of these primers are discussed below.

#### ‘iap’-based primers ELMIAPF/R

For this investigation, we had specifically chosen ‘*iap*’ gene common to all members of the genus *Listeria* as target because the comparison of all ‘*iap*’ genes has indicated that there were conserved regions at 5′ and 3′ ends, while the internal portions are highly specific (Bubert et al. 1992). The ‘*iap*’ gene of *L. monocytogenes* encodes the major extracellular protein (P60) (Kuhn and Goebel 1989), which has been shown to be basically an essential murein hydrolase required for adherence/invasion of the organism to the targeted eucaryotic cell. It has been recently shown that the corresponding *iap* gene portion is also hypervariable in length in different isolates belonging to the same serotypes, thereby, can help in identification of different strains of *L. monocytogenes.*

In order to delineate the species identity of *Listeria*spp., some *Listeria monocytogenes*-specific primers targeted against selected virulence genes were initially explored individually in the study for PCR assays. The first primer pair selected for the purpose was targeted against ‘*iap*’ gene as used previously by Klein and Juneja (1997). The PCR amplification conditions were exactly the same as indicated above for *16S rRNA-*based PCR assay. Agarose gel picture as shown in Fig. 1 (Lanes 4 and 5) revealed a PCR amplified product of 371 bp size with template DNA from both the strains of *Listeria monocytogenes* used in the study. In order to explore the possibility of confirming the authenticity of PCR products (371 bp) of above ‘*iap*’-based primers (external) specific for *Listeria monocytogenes*, a pair of primers targeted against internal region of ‘*iap*’ gene with the amplified PCR product was also tested in this study. The template used in the PCR assay based on internal ‘*iap*’ primers was the amplified 371 bp product obtained from the previous PCR assay. The PCR assay using internal ‘*iap*’ primers and the amplified product of the external ‘*iap*’-based PCR assay resulted into the amplification of 119 bp product as can be evidenced from Fig. 1 (Lanes 7 and 8). However, two additional non-specific bands were also detected on the gel albeit at a relatively low intensity. One such band corresponds with the 371 bp product of external ‘*iap*’-based PCR indicating the possible carry over of the template DNA. The nested PCR conditions used in the study were optimized that resulted into 119 bp product only using 0.5 μl of 1:10 diluted PCR amplified product from external *iap* primers (data not shown). Our results pertaining to amplification of the targeted DNA with ‘*iap*’-based primers are in close agreement with those of Klein and Juneja (1997) who had previously used there primers for RT-PCR instead of direct PCR with the sole objective of detecting viable cells of *L. monocytogenes.* The RT-PCR assay developed by these investigators could amplify a 371 bp product with ELM*IAP*F/R from only the viable cells when cDNA synthesized from mRNA of *L. monocytogenes* was used as the template. However, in our study these primers were intended to amplify *L. monocytogenes* template DNA through direct PCR for subsequent application of the assay in detection of the targeted organism in raw milk and paneer which do not require any harsh processing treatments. Our results with regard to the use of internal primers-based ‘*iap*’ gene are also comparable to those of Klein and Juneja (1997), although the purpose of using these primers in the two studies was different. Klein and Juneja (1997) had used this internal set of primers just to produce a probe for confirmation of their RT-PCR results for detection of viable *L. monocytogenes*. However, we have used these primers for confirming the specificity of 371 bp product.

#### ‘iap’-based primers (Mono A and Lis 1B)

Another set of primers targeted against a different region of ‘*iap*’ was also included in our study for determining their possible application in detection of *Listeria monocytogenes* by PCR assay. The PCR assay set up with this primer pair Mono A and Lis 1B could amplify a 660 bp product with template from *Listeria monocytogenes*. Lis 1 set of primers in the assay worked reasonably well with the PCR amplification parameters as used for other ‘*iap*’/’*Prf*A’-based PCR assays (data not shown). Our PCR results obtained with Mono A and Lis1B set of primers are consistent with the observations recorded by Bubert et al. (1999) who could also get the amplification of the 660 bp product with this set of primers and exploited it for development of a multiplex PCR assay for *L. monocytogenes*.

#### ‘prfA’-based primers ELMPRFF/R

Since the virulence genes in *Listeria monocytogenes* are coregulated by ‘*prf*A’ gene which codes for a 27.1 kDa protein (Chakraborty et al. 1992) which positively regulate all the virulence genes, this gene can also be targeted for detection of *L. monocytogenes* by PCR. We also explored ‘*prf*A’ in the PCR assay using a pair of primers which resulted into the amplification of 508 bp product as seen in Fig. 1 (Lanes 10 and 11). Our results in this regard are again exactly compatible with those of Klein and Juneja (1997) who could also obtained 508 bp products in RT-PCR-based assay used for detection of *L. monocytogenes*.

### Primers targeted against ‘*hly*’ gene

*Listeria monocytogenes* is also capable of producing hemolysins which are involved in the lysis of vacuole and the erythrocytes. The main factor involved in the lysis of the vacuole is the protein that has pore forming activity. ‘Listeriolysin O’, a 58 kDa protein, is encoded on ‘*hly A*’ gene. ‘*Hly A*’ gene has also been targeted for development of PCR-based assays intended for detection of *L. monocytogenes*. In this study, we used three sets of primers targeted against different regions of ‘*hly*’ gene of *Listeria monocytogenes* as used previously by other workers and evaluated their efficacy in their respective PCR assays. The results pertaining to the same have been presented in Fig. 2.

#### ‘hly’-based primers ELMHLYF/R

The primer pair namely ELM*HLY*F/R targeted against ‘*hly*’ gene when used in the PCR assay produced an amplified product of 713 bp with template DNA from *Listeria monocytogenes* (Fig. 2, Lanes 1 and 2). The PCR amplification conditions were exactly the same as those used for 16S rRNA-, ‘*iap*’- and ‘*Prf*A’-based PCR assays. Here also to further check the authenticity of the 713 bp product obtained with *hly*-based primer set, another pair of primers namely ILM*HLY*F/R representing the internal region of 713 bp amplified product was also tested separately in a different PCR assay using the 713 bp amplified product as template and the same amplification parameters as described for *hly*-based PCR assay. This resulted into the amplification of 188 bp product falling within the internal region of 713 bp product as shown in Fig. 2 (Lanes 4 and 5). However, two more additional non-specific bands one corresponding with 713 bp product along with a smaller band could also be detected on the agarose gel. The non-specific bands could be eliminated by following the steps as described previously. Our findings in this regard are further supported by similar observations made by Klein and Juneja (1997) who could also achieve a 713 bp product with their RT-PCR assay using ELM*HLY*F/R set of primers and 188 bp product with ILM*HLY*F/R primers. In this particular case also, these investigators used the internal primers to develop a *L. monocytogenes*-specific probe. On the other hand, the use of these internal primers in our study was intended to confirm the identity of 713 bp product from *L. monocytogenes* template.

#### *‘hly*A’-based primers Hly1/2

Another primer pair targeted against ‘*hly*A’ gene when subjected to PCR with the template from *Listeria monocytogenes* yielded an amplified DNA band of 276 bp as can be revealed from Fig. 2 (Lanes 7 and 8). The PCR conditions used in the assay were more or less similar to those used for the other above-mentioned PCR assays except that an annealing temperature of 55 °C was used in place of 60 °C. The results pertaining to efficacy of ‘*hly A*’ gene-based primers with template from *L. monocytogenes* in the PCR assay used in this study are in close agreement with those of Cooray et al. (1994) who could also detect a 276 bp amplified product in their multiplex PCR assay where they had combined the primers targeted against ‘*hly A*’ gene with those from ‘*prf*A’ and ‘plcB’ genes. However, the PCR cycling conditions used in the two studies were slightly different.

#### ‘*hly* K9’-based primers HF9/HR9

Since K-9 region derived from the non-coding region of ‘*hly*’ gene has been found to be highly polymorphic, this particular region can also be explored for developing a PCR assay for distinguishing different strains of *L. monocytogenes*. This type of assay can be extensively valuable for epidemiological typing. The PCR assay based on *hly K9* set of primers produced an amplified PCR product of 384 bp with *Listeria monocytogenes* template. The PCR results pertaining to the same have been recorded in Fig. 2 (Lanes 10 and 11). Our results in this regard can be substantiated by similar observations made by Wagner et al. (2000) who also recorded the amplification of 384 bp product in their PCR-based study only when *L. monocytogenes* template was used in the assay.

A critical appraisal of overall PCR results obtained with the respective sets of primers in this study clearly indicates that all the primers worked quite reasonably even under same PCR cycling conditions and hence, there is a possibility of using them simultaneously in conjunction for possible development of a multiplex PCR assay which could authentically detect *L. monocytogenes* in dairy foods rapidly.

### Development of multiplex PCR using different combinations of primers for detection of *Listeria monocytogenes*

**Fig. 3:**
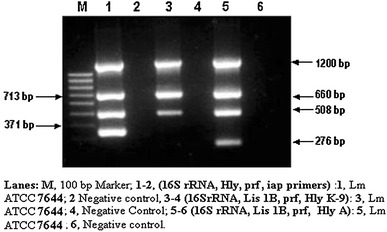
Evaluation of different combinations of primers targeted against different genes for detection of *Listeria* spp. and *Listeria monocytogenes* by respective multiplex PCR assays

After evaluation of individual sets of primers in their respective PCR assays, an attempt was then made to explore different combination of these primers to develop a reliable multiplex PCR assay for detection of *Listeria* as such or more specifically *Listeria monocytogenes*. Initially, we tried three combinations involving four different sets of primers in three different multiplex PCR assays using identical PCR parameters. Combination 1 included primer sets targeted against *16S rRNA*, ‘*hly*’, ‘*prf*A’, ‘*iap*’ genes, Combination 2 comprised of primers targeted against *16S rRNA, ‘Lis 1B’*, ‘*prf*A’, ‘*hly K9*′ and Combination 3 used primer pairs targeted against *16S rRNA, ‘Lis 1B’*, ‘*prf*A’ and ‘*hly*A’. The PCR parameters used included initial denaturation at 95 °C for 4 min followed by 35 cycles each of denaturation at 94 °C for 30 s, annealing at 58 °C for 1 min and extension at 72 °C for 1 min and an additional step of extended extension at 72 °C for 10 min. The results pertaining to the same have been presented in Fig. 3. As is quite evident from the banding patterns revealed on agarose gel, the multiplex PCR assay set up with primers based on *16S rRNA*, *hly*, *prf*A and *iap* (combination 1) amplified the targeted DNA specifically producing four distinct bands of 1,200, 713, 508, 371 bp, respectively (Fig. 3, Lane 1), which matched exactly the same with the PCR products obtained with individual primer pairs as described previously. The multiplex PCR with combination 2-based primers could amplify *L. monocytogenes* template resulting into formation of 1,200, 660 and 508 bp bands, respectively, representing *16S rRNA, Lis 1B and prfA genes* (Fig. 3, Lane 3). However, the fourth expected amplified PCR product of size 384 bp representing *hly K9* could not be detected on the agarose gel. This discrepancy could possibly be attributed to the use of high annealing temperature in the multiplex PCR which may not be suitable for amplification of K-9 fragment. The combination 3 on the other hand again resulted into the amplification of all the four targeted genes in their respective multiplex PCR assay as indicated by the formation of 1,200, 660, 508 and 276 bp bands on the gel (Fig. 3, Lane 5). A comparative evaluation of the three combinations of the primers in their respective multiplex PCR assays clearly indicates that combination 1 and combination 3 performed reasonably well in their respective multiplex PCR assays and the performance of combination 2 was comparatively lower due to non amplification of 384 bp product. Hence, the latter was not considered for further study. Out of the above combinations, 1 and 3 were selected for further improvements in the study.

The main purpose of developing a multiplex PCR in this study was to minimize the possibility of missing *L. monocytogenes* from detection. With this objective in mind, we had combined four sets of primers including one targeted against genus-specific *16S rRNA* and the other three targeted against different regions of virulence genes ‘*iap*’, ‘*hly*’ and ‘*prf*A’ for simultaneous amplification of template DNA from *L. monocytogenes*. Our results in this regard are consistent with similar findings made by Cooray et al. (1994) who also combined three sets of primers targeted against *hly A*, *prf* A and *plc B* genes in their multiplex PCR assay which could amplify specifically 795, 571 and 276 bp products with *L. monocytogenes* only. It is quite plausible that some *L. monocytogenes* may lack one or more virulence determinants either because of some mutations or inability of some genes to express under certain conditions. Though the frequency of spontaneous mutations or deletions in these virulence associated genes is not precisely known as yet, we cannot deny the possibility that some mutants like those produced by transposon insertions may exist in nature (Kathariou et al. 1987; Sun et al. 1990) as has been proposed by Cooray et al. (1994). Therefore, it seemed logical and relevant to develop a procedure in which various virulence associated genes could be detected simultaneously in a single step. Working on similar lines, Klein and Juneja (1997) made an attempt to use three pairs of primers targeted against ‘*hly*’, ‘*iap* ‘and ‘*prf*A’ genes simultaneously in their RT-PCR assay to rule out the possibility of false positive results in PCR-based assays for detection of viable *L. monocytogenes*. However, they were not able to get amplification of all the targeted genes consistently with stronger signal achieved only with primer pair targeted against ‘*iap*’ gene, thereby, greatly limiting the applicability of such multiplex RT-PCR assay.

### Optimization of multiplex PCR based on combination 3

 While further improving the multiplex PCR in terms of using different concentrations of Taq polymerase, dNTPs, primers, manganese chloride, annealing time and number of amplification cycles, it was experienced that two of the amplified products viz. 508 and 276 bp were quite inconsistent in the multiplex PCR and many times either could not be detected on the agarose gel or produced diminished bands (data not shown) that are unable to be visualized. However, the two products namely 1,200 bp based on *16S rRNA*, 713 bp with *ELMHLYF/R* and 660 bp based on *Lis 1B* could always be detected in the multiplex PCR. This inconsistency in the behavior of some primers when used in combination in the multiplex PCR is not an unusual phenomenon due to possible structural interactions between different pieces of oligomers. Although this contention cannot be substantiated as yet, the unequal level of amplifications observed by Klein and Juneja (1997) in their RT-PCR assay using primers targeted against ‘*iap*’, ‘*prf* A’ and ‘*hly*’ genes may indirectly explain the reason for this discrepancy.

In the light of these observations, we finally resorted to develop a multiplex PCR based on two sets of primers one specific for *Listeria* (*16S rRNA*) and another for *Listeria monocytogenes* (‘*hly*’) for eventual application in dairy foods.

### Multiplex PCR based on two sets of primers for detection of *Listeria monocytogenes*

**Fig. 4:**
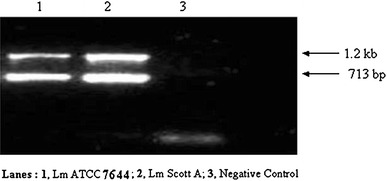
Multiplex PCR assay using two pairs of primers targeted against *16S rRNA* and *Hly*

Since difficulties were encountered in terms of amplification of all the expected products during optimization of a multiplex PCR based on simultaneous use of four sets of primers, efforts were then directed to develop a multiplex PCR assay exploring only two sets of primers i.e. one genus specific and second *Listeria monocytogenes* specific to get consistent and reproducible results. Two such selected primer pairs which were targeted against *16S rRNA* (genus-specific, 1,200 bp PCR product) and ‘*hly*’ (*Listeria monocytogenes-*specific, 713 bp PCR product) since these two primer pairs consistently amplified the specific product. Our previous trials during standardization using the PCR amplification conditions of initial denaturation at 95 °C/4 min followed by 35 cycles each of denaturation at 95 °C/30 s, annealing at 60 °C/1 min and extension at 72 °C/1 min followed by final extension at 72 °C for 5 min led to the amplification of 1,200 bp product specific for *16S rRNA* gene of *Listeria* and 713 bp product specific for *hly* gene of *Listeria monocytogenes* (Fig. 4, Lanes 1 and 2). The multiplex PCR based on *16S rRNA* and ‘*hly*’ genes was successfully applied to selected spiked and natural market dairy food samples for detection of *Listeria monocytogenes* that forms the subject of a separate communication.

From the foregoing presentation, it can be concluded that the multiplex PCR assay developed with two sets of primers targeted against *16S rRNA* and ‘*hly*’ genes could be extremely valuable and of considerable practical utility in dairy industry for monitoring dairy foods for *L. monocytogenes* and hence could help in protecting the health of the consumers against this high-risk food pathogen.
